# Supplementary material for: Adolescent Awkwardness: Alterations in Temporal Control Characteristics of Posture with Maturation and the Relation to Movement Exploration
Source: Brain Sci. 2020 Apr 5;10(4):216. doi: 10.3390/brainsci10040216 (PMC7226109; doi:10.3390/brainsci10040216)
Supplement: Supplementary file 1 [file brainsci-10-00216-s001.zip › brainsci-763104-supplementary/File_S1.pdf]

Anthropometric Differences

The anthropometrical properties as presented in Table 1 differed overall significantly between the three groups, seen in age $F(2,8.083) = 118.158$, $p < 0.001$, $\eta^2 = 0.911$; height $F(2,13.656) = 24.800$, $p < 0.001$, $\eta^2 = 0.683$ and weight $F(2,13.836) = 25.198$, $p < 0.001$, $\eta^2 = 0.687$. Post-Hoc tests confirmed that adults were taller, heavier and older than adolescents, regardless of whether the adults were smaller than 180cm (height: $p = 0.007$, weight: $p = 0.001$; age $p < 0.001$) or taller than 190cm (height: $p < 0.001$, weight: $p < 0.001$; age $p < 0.001$). The two adult groups differed significantly in height ($p = 0.002$) and weight ($p = 0.013$) but not in age ($p = 0.353$).
